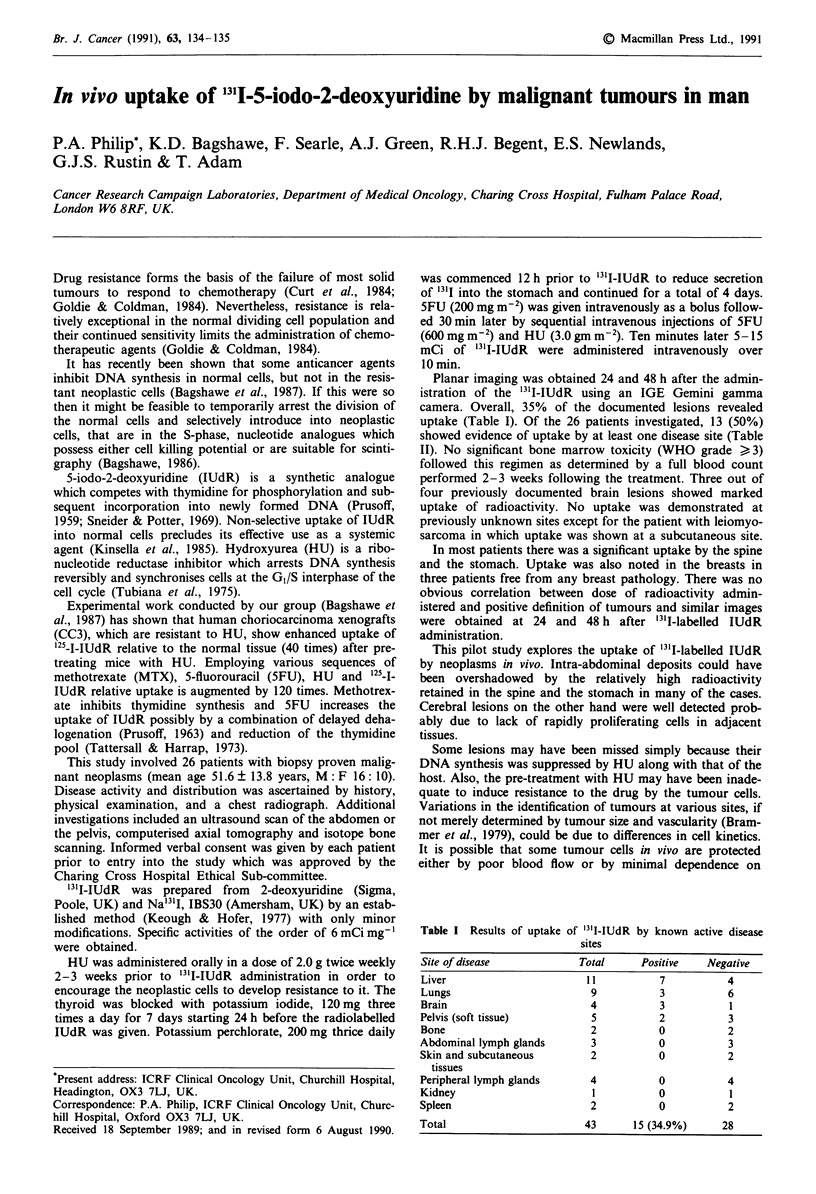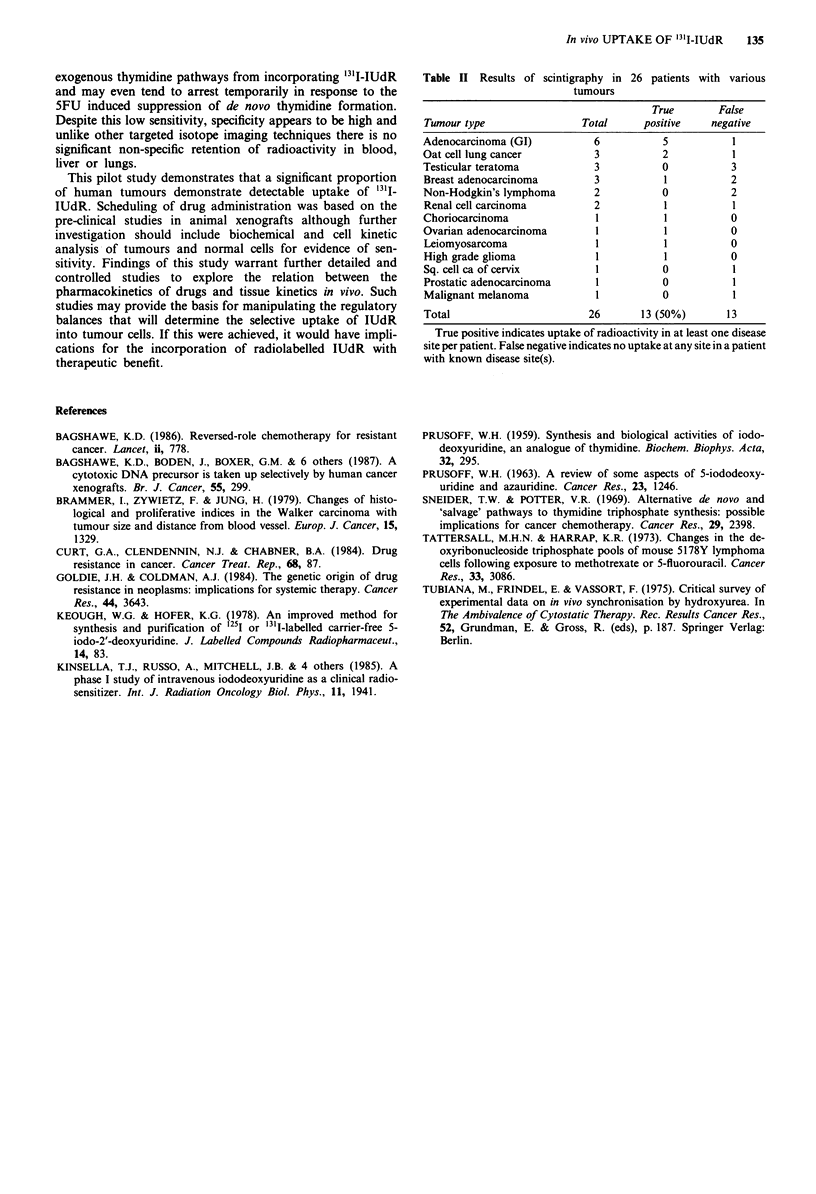# In vivo uptake of 131I-5-iodo-2-deoxyuridine by malignant tumours in man.

**DOI:** 10.1038/bjc.1991.27

**Published:** 1991-01

**Authors:** P. A. Philip, K. D. Bagshawe, F. Searle, A. J. Green, R. H. Begent, E. S. Newlands, G. J. Rustin, T. Adam

**Affiliations:** Department of Medical Oncology, Charing Cross Hospital, London, UK.


Br. J. Cancer (1991), 63, 134-135 © Macmillan Press Ltd., 1991

### *In vivo* uptake of $^{131}$I-5-iodo-2-deoxyuridine by malignant tumours in man

P.A. Philip*, K.D. Bagshawe, F. Searle, A.J. Green, R.H.J. Begent, E.S. Newlands, G.J.S. Rustin & T. Adam

*Cancer Research Campaign Laboratories, Department of Medical Oncology, Charing Cross Hospital, Fulham Palace Road, London W6 8RF, UK.*

Drug resistance forms the basis of the failure of most solid tumours to respond to chemotherapy (Curt *et al.*, 1984; Goldie & Coldman, 1984). Nevertheless, resistance is relatively exceptional in the normal dividing cell population and their continued sensitivity limits the administration of chemotherapeutic agents (Goldie & Coldman, 1984).

It has recently been shown that some anticancer agents inhibit DNA synthesis in normal cells, but not in the resistant neoplastic cells (Bagshawe *et al.*, 1987). If this were so then it might be feasible to temporarily arrest the division of the normal cells and selectively introduce into neoplastic cells, that are in the S-phase, nucleotide analogues which possess either cell killing potential or are suitable for scintigraphy (Bagshawe, 1986).

5-iodo-2-deoxyuridine (IUdR) is a synthetic analogue which competes with thymidine for phosphorylation and subsequent incorporation into newly formed DNA (Prusoff, 1959; Sneider & Potter, 1969). Non-selective uptake of IUdR into normal cells precludes its effective use as a systemic agent (Kinsella *et al.*, 1985). Hydroxyurea (HU) is a ribonucleotide reductase inhibitor which arrests DNA synthesis reversibly and synchronises cells at the $G_1$/S interphase of the cell cycle (Tubiana *et al.*, 1975).

Experimental work conducted by our group (Bagshawe *et al.*, 1987) has shown that human choriocarcinoma xenografts (CC3), which are resistant to HU, show enhanced uptake of $^{125}$-I-IUdR relative to the normal tissue (40 times) after pretreating mice with HU. Employing various sequences of methotrexate (MTX), 5-fluorouracil (5FU), HU and $^{125}$-I-IUdR relative uptake is augmented by 120 times. Methotrexate inhibits thymidine synthesis and 5FU increases the uptake of IUdR possibly by a combination of delayed dehalogenation (Prusoff, 1963) and reduction of the thymidine pool (Tattersall & Harrap, 1973).

This study involved 26 patients with biopsy proven malignant neoplasms (mean age 51.6 ± 13.8 years, M : F 16 : 10). Disease activity and distribution was ascertained by history, physical examination, and a chest radiograph. Additional investigations included an ultrasound scan of the abdomen or the pelvis, computerised axial tomography and isotope bone scanning. Informed verbal consent was given by each patient prior to entry into the study which was approved by the Charing Cross Hospital Ethical Sub-committee.

$^{131}$I-IUdR was prepared from 2-deoxyuridine (Sigma, Poole, UK) and Na$^{131}$I, IBS30 (Amersham, UK) by an established method (Keough & Hofer, 1977) with only minor modifications. Specific activities of the order of 6 mCi mg$^{-1}$ were obtained.

HU was administered orally in a dose of 2.0 g twice weekly 2-3 weeks prior to $^{131}$I-IUdR administration in order to encourage the neoplastic cells to develop resistance to it. The thyroid was blocked with potassium iodide, 120 mg three times a day for 7 days starting 24 h before the radiolabelled IUdR was given. Potassium perchlorate, 200 mg thrice daily was commenced 12 h prior to $^{131}$I-IUdR to reduce secretion of $^{131}$I into the stomach and continued for a total of 4 days. 5FU (200 mg m$^{-2}$) was given intravenously as a bolus followed 30 min later by sequential intravenous injections of 5FU (600 mg m$^{-2}$) and HU (3.0 gm m$^{-2}$). Ten minutes later 5-15 mCi of $^{131}$I-IUdR were administered intravenously over 10 min.

Planar imaging was obtained 24 and 48 h after the administration of the $^{131}$I-IUdR using an IGE Gemini gamma camera. Overall, 35% of the documented lesions revealed uptake (Table I). Of the 26 patients investigated, 13 (50%) showed evidence of uptake by at least one disease site (Table II). No significant bone marrow toxicity (WHO grade ≥3) followed this regimen as determined by a full blood count performed 2-3 weeks following the treatment. Three out of four previously documented brain lesions showed marked uptake of radioactivity. No uptake was demonstrated at previously unknown sites except for the patient with leiomyosarcoma in which uptake was shown at a subcutaneous site.

In most patients there was a significant uptake by the spine and the stomach. Uptake was also noted in the breasts in three patients free from any breast pathology. There was no obvious correlation between dose of radioactivity administered and positive definition of tumours and similar images were obtained at 24 and 48 h after $^{131}$I-labelled IUdR administration.

This pilot study explores the uptake of $^{131}$I-labelled IUdR by neoplasms *in vivo*. Intra-abdominal deposits could have been overshadowed by the relatively high radioactivity retained in the spine and the stomach in many of the cases. Cerebral lesions on the other hand were well detected probably due to lack of rapidly proliferating cells in adjacent tissues.

Some lesions may have been missed simply because their DNA synthesis was suppressed by HU along with that of the host. Also, the pre-treatment with HU may have been inadequate to induce resistance to the drug by the tumour cells. Variations in the identification of tumours at various sites, if not merely determined by tumour size and vascularity (Brammer *et al.*, 1979), could be due to differences in cell kinetics. It is possible that some tumour cells *in vivo* are protected either by poor blood flow or by minimal dependence on

**Table I** Results of uptake of $^{131}$I-IUdR by known active disease sites

| *Site of disease* | *Total* | *Positive* | *Negative* |
|---|---|---|---|
| Liver | 11 | 7 | 4 |
| Lungs | 9 | 3 | 6 |
| Brain | 4 | 3 | 1 |
| Pelvis (soft tissue) | 5 | 2 | 3 |
| Bone | 2 | 0 | 2 |
| Abdominal lymph glands | 3 | 0 | 3 |
| Skin and subcutaneous tissues | 2 | 0 | 2 |
| Peripheral lymph glands | 4 | 0 | 4 |
| Kidney | 1 | 0 | 1 |
| Spleen | 2 | 0 | 2 |
| Total | 43 | 15 (34.9%) | 28 |

*Present address: ICRF Clinical Oncology Unit, Churchill Hospital, Headington, OX3 7LJ, UK.
Correspondence: P.A. Philip, ICRF Clinical Oncology Unit, Churchill Hospital, Oxford OX3 7LJ, UK.
Received 18 September 1989; and in revised form 6 August 1990.

exogenous thymidine pathways from incorporating $^{131}$I-IUdR and may even tend to arrest temporarily in response to the 5FU induced suppression of *de novo* thymidine formation. Despite this low sensitivity, specificity appears to be high and unlike other targeted isotope imaging techniques there is no significant non-specific retention of radioactivity in blood, liver or lungs.

This pilot study demonstrates that a significant proportion of human tumours demonstrate detectable uptake of $^{131}$I-IUdR. Scheduling of drug administration was based on the pre-clinical studies in animal xenografts although further investigation should include biochemical and cell kinetic analysis of tumours and normal cells for evidence of sensitivity. Findings of this study warrant further detailed and controlled studies to explore the relation between the pharmacokinetics of drugs and tissue kinetics *in vivo*. Such studies may provide the basis for manipulating the regulatory balances that will determine the selective uptake of IUdR into tumour cells. If this were achieved, it would have implications for the incorporation of radiolabelled IUdR with therapeutic benefit.

**Table II** Results of scintigraphy in 26 patients with various tumours

| *Tumour type* | *Total* | *True positive* | *False negative* |
|---|---|---|---|
| Adenocarcinoma (GI) | 6 | 5 | 1 |
| Oat cell lung cancer | 3 | 2 | 1 |
| Testicular teratoma | 3 | 0 | 3 |
| Breast adenocarcinoma | 3 | 1 | 2 |
| Non-Hodgkin's lymphoma | 2 | 0 | 2 |
| Renal cell carcinoma | 2 | 1 | 1 |
| Choriocarcinoma | 1 | 1 | 0 |
| Ovarian adenocarcinoma | 1 | 1 | 0 |
| Leiomyosarcoma | 1 | 1 | 0 |
| High grade glioma | 1 | 1 | 0 |
| Sq. cell ca of cervix | 1 | 0 | 1 |
| Prostatic adenocarcinoma | 1 | 0 | 1 |
| Malignant melanoma | 1 | 0 | 1 |
| Total | 26 | 13 (50%) | 13 |

True positive indicates uptake of radioactivity in at least one disease site per patient. False negative indicates no uptake at any site in a patient with known disease site(s).

### References

BAGSHAWE, K.D. (1986). Reversed-role chemotherapy for resistant cancer. *Lancet*, **ii**, 778.

BAGSHAWE, K.D., BODEN, J., BOXER, G.M. & 6 others (1987). A cytotoxic DNA precursor is taken up selectively by human cancer xenografts. *Br. J. Cancer*, **55**, 299.

BRAMMER, I., ZYWIETZ, F. & JUNG, H. (1979). Changes of histological and proliferative indices in the Walker carcinoma with tumour size and distance from blood vessel. *Europ. J. Cancer*, **15**, 1329.

CURT, G.A., CLENDENNIN, N.J. & CHABNER, B.A. (1984). Drug resistance in cancer. *Cancer Treat. Rep.*, **68**, 87.

GOLDIE, J.H. & COLDMAN, A.J. (1984). The genetic origin of drug resistance in neoplasms: implications for systemic therapy. *Cancer Res.*, **44**, 3643.

KEOUGH, W.G. & HOFER, K.G. (1978). An improved method for synthesis and purification of $^{125}$I or $^{131}$I-labelled carrier-free 5-iodo-2'-deoxyuridine. *J. Labelled Compounds Radiopharmaceut.*, **14**, 83.

KINSELLA, T.J., RUSSO, A., MITCHELL, J.B. & 4 others (1985). A phase I study of intravenous iododeoxyuridine as a clinical radiosensitizer. *Int. J. Radiation Oncology Biol. Phys.*, **11**, 1941.

PRUSOFF, W.H. (1959). Synthesis and biological activities of iododeoxyuridine, an analogue of thymidine. *Biochem. Biophys. Acta*, **32**, 295.

PRUSOFF, W.H. (1963). A review of some aspects of 5-iododeoxyuridine and azauridine. *Cancer Res.*, **23**, 1246.

SNEIDER, T.W. & POTTER, V.R. (1969). Alternative *de novo* and 'salvage' pathways to thymidine triphosphate synthesis: possible implications for cancer chemotherapy. *Cancer Res.*, **29**, 2398.

TATTERSALL, M.H.N. & HARRAP, K.R. (1973). Changes in the deoxyribonucleoside triphosphate pools of mouse 5178Y lymphoma cells following exposure to methotrexate or 5-fluorouracil. *Cancer Res.*, **33**, 3086.

TUBIANA, M., FRINDEL, E. & VASSORT, F. (1975). Critical survey of experimental data on *in vivo* synchronisation by hydroxyurea. In *The Ambivalence of Cytostatic Therapy. Rec. Results Cancer Res.*, **52**, Grundman, E. & Gross, R. (eds), p. 187. Springer Verlag: Berlin.